# Energy Exchange and Coherent Behavior of Monolayer TMD Polaritonic Systems in All-Dielectric Metasurfaces Supporting Quasi-Bound States in the Continuum

**DOI:** 10.3390/nano16191197

**Published:** 2026-09-22

**Authors:** Jiani Li, Tao Jiang, Ze Peng, Ze Li, Meng Wang

**Affiliations:** 1 Research Center for Quantum Physics and Technologies, School of Physical Science and Technology, Inner Mongolia University, Hohhot 010021, China; 2 Key Laboratory of Semiconductor Photovoltaic Technology and Energy Materials at Universities of Inner Mongolia Autonomous Region, Inner Mongolia University, Hohhot 010021, China

**Keywords:** strong light-matter coupling, quasi-bound states in the continuum (q-BICs), transition metal dichalcogenides (TMDs)

## Abstract

Room-temperature strong light–matter coupling in two-dimensional transition metal dichalcogenides (TMDs) is crucial for the development of polaritonic devices. In this work, we construct an all-dielectric metasurface platform supporting quasi-bound states in the continuum (q-BICs) and achieve strong coupling with four representative monolayer TMDs (MoSe_2_, MoS_2_, WS_2_, and WSe_2_) through tailored geometric scaling for zero-detuning matching. Based on the coupled oscillator model (COM) and the Heisenberg–Langevin model, we systematically analyze the coupling characteristics and polaritonic dynamics of these customized hybrid systems. The results show that the tailored MoSe_2_-cavity system achieves the maximum coupling strength (36.8 meV) and the fastest energy exchange, whereas the customized WSe_2_-cavity system demonstrates optimal coherence stability enabled by the highest cooperativity (*C* = 3.50). This study elucidates how the synergistic interplay between the materials’ excitonic properties and the customized photonic environments decouples the energy exchange rate from coherence stability. These findings establish a practical configuration design framework for room-temperature polaritonic devices, paving the way for targeted applications that require either ultrafast responses or sustained polaritonic coherence.

## 1. Introduction

All-dielectric metasurfaces exhibit low optical losses, high-quality-factor resonances, and excellent electromagnetic field localization capabilities, making them ideal platforms for enhancing light–matter interactions [1,2,3,4,5,6]. Among these, bound states in the continuum (BICs) represent a class of localized dark states decoupled from the radiation continuum, theoretically possessing an infinite quality factor [1,2,3,4,7,8,9]. To facilitate controllable coupling with the far field in practical implementations, ideal BICs are typically transformed into quasi-bound state in the continuum (q-BIC) modes with finite Q factors by introducing asymmetry [7,8,9]. These quasi-bound modes can generate strong electromagnetic field enhancements and tightly confined near-field distributions, providing a robust platform for realizing strong light–matter coupling [1,2,3,4,7,8,9].

Exciton–polariton strong coupling represents an important frontier in the field of light–matter interactions, exhibiting unique physical phenomena and modulation potential in the visible to near-infrared spectrum [10,11,12,13,14,15,16]. When the electromagnetic field of the cavity mode matches the intrinsic exciton transition of the two-dimensional material in both space and frequency, and their coupling strength g exceeds the total energy dissipation rate of the system, the system enters the strong coupling regime, thereby forming a new light-exciton hybrid state (polariton) [17]. The strong coupling criterion can be expressed as [18,19,20]:

(1) $$g>\sqrt{\frac{\gamma_{Q}^{2}+\gamma_{m}^{2}}{2}}.$$

Here, $\gamma_{Q}$ and $\gamma_{m}$ represent the linewidths of the cavity mode and the exciton mode, respectively. Upon satisfying this criterion, continuous coherent energy exchange occurs between the cavity mode and the exciton mode, splitting the original single-resonance peak into upper and lower polariton branches, which manifests as typical Rabi splitting in the transmission or reflection spectra [21,22,23,24,25,26,27]. Strongly coupled systems provide a highly promising platform for investigating Bose–Einstein condensation and developing novel polaritonic devices such as low-threshold lasers, ultrafast optical switches, and quantum information processors [24,25,26,27,28,29,30,31,32,33].

Although previous pioneering studies have successfully demonstrated strong coupling between TMD excitons and optical q-BICs using grating-based asymmetric metasurfaces [34], existing investigations predominantly focus on isolated, single-material systems. Consequently, there remains a lack of systematic evaluation across different spectral bands [32,35]. Realizing precise zero-detuning across diverse spectral bands necessitates customized structural scaling, yet the macroscopic polaritonic consequences of such scaling have not been fully elucidated. To address this, we establish a comparative framework encompassing four distinct tailored TMD–cavity configurations. Ultimately, this systematic evaluation enables us to identify the optimal hybrid synergies for specific functional demands: the tailored MoSe_2_ system maximizes the ultrafast energy exchange rate, whereas the customized WSe_2_ configuration delivers superior polaritonic coherence stability. These findings provide a highly practical material-selection and structural design guide for versatile room-temperature polaritonic devices.

In this work, we construct an all-dielectric metasurface platform supporting q-BICs and investigate four tailored hybrid systems incorporating representative monolayer TMDs (MoSe_2_, MoS_2_, WS_2_, and WSe_2_). To strictly satisfy the zero-detuning condition, the geometric parameters of the metasurfaces are individually customized for each material. Based on the coupled oscillator model (COM) and the quantum Heisenberg–Langevin model, our analytical results indicate that the macroscopic coupling performance is a synergistic result of intrinsic material properties and customized photonic environments. Specifically, the MoSe_2_ hybrid system exhibits the strongest coupling strength with an ultrafast Rabi exchange period of 56 fs, while the WSe_2_ hybrid system demonstrates superior coherence preservation (cooperativity *C* = 3.50). These results establish a comprehensive framework for selecting and designing robust polaritonic systems tailored for specific room-temperature photonic and quantum applications.

## 2. Results and Discussion

Using Lumerical FDTD, we construct an all-dielectric metasurface composed of a TiO_2_ on SiO_2_ composite substrate and an array of tilted silicon elliptical cylinders, as shown in Figure 1a. During the simulations, the optical constants of SiO_2_ and Si are directly taken from the standard datasets in the built-in material library of Lumerical FDTD. For the TiO_2_ layer, we use the experimentally measured and fitted thin-film optical parameters reported by Sarkar et al. [36]. Periodic boundary conditions are applied in the *x* and *y* directions of the unit cell, while perfectly matched layers (PMLs) are employed in the *z* direction. Figure 1b illustrates the geometric parameters of the unit cell in the *x*–*y* plane, where the major and minor semi-axes of the silicon elliptical cylinder are *a* and *b*, respectively, and the unit cell periods in the *X* and *Y* directions are ${\;P}_{1\;}$ and $P_{2}$, respectively. The asymmetry parameter is defined as α=sinθ, where θ represents the angle between the major axis of the rod and the *y*-axis. Taking the MoSe_2_ hybrid system as a representative example, the customized geometric parameters to ensure precise spectral matching are optimized as $P_{1}=610$, $P_{2}=450$, a=209, b=58, pillar height h=60, $\theta =30^{{}^{\circ}}$, and an inter-ellipse distance of 300 (all length units in nm). Determined by their band structures, the intrinsic room-temperature transition energies of monolayer MoSe_2_, MoS_2_, WS_2_, and WSe_2_ exhibit significant differences. Consequently, implementing a uniform microcavity geometry across diverse TMD materials would induce severe energy detuning, thereby hindering the formation of polaritons. To strictly satisfy the zero-detuning condition ($\omega_{Q}=\omega_{m}$) required for strong coupling, the structural dimensions and asymmetry parameters were individually optimized for each specific TMD (see Table S1). Given that such geometric adaptations inevitably modulate the local electromagnetic environment, specifically the mode volume and near-field enhancement, our analysis investigates the exciton–photon coupling dynamics as a synergistic outcome of the integrated hybrid systems. This holistic approach provides a comprehensive evaluation of polaritonic performance, offering practical physical insights for the structural design and material selection of future polaritonic technologies.

To clarify the physical origin of the resonance mode, a multipole expansion was performed taking the resonance peak of the MoSe_2_ cavity as an example [37,38], quantitatively characterizing the contribution of each electromagnetic component to the resonance. Here, the electric dipole $P_{\alpha }$, magnetic dipole $M_{\alpha }$, toroidal dipole $T_{\alpha }$, electric quadrupole $Q_{\alpha \beta }^{\left(e\right)}$, and magnetic quadrupole $Q_{\alpha \beta }^{\left(m\right)}$ are denoted as *P*, *M*, *T*, *Q_e_*, and *Q_m_*, respectively (calculation details are provided in the Supporting Information) [39,40,41]. As shown in Figure 1e, the scattering power at the resonance energy is dominated by the magnetic dipole. Furthermore, the near-field distribution in Figure 1f reveals that the electric field vectors form highly localized closed electromagnetic vortices inside the silicon pillar (see Figure S1 for other cavities), which is highly consistent with the previous mode decomposition. Such a magnetic-dipole-dominated vortex field is a signature topological feature of q-BICs [42]. Further analysis of the evolution mapping of the asymmetry parameter θ shown in Figure 1c indicates that there is no energy leakage in the absence of structural asymmetry. Upon introducing the asymmetry parameter, the rotational symmetry is broken, leading to energy leakage that can be observed in the reflection spectrum. Additionally, Figure 1d investigates the relationship between the asymmetry and the *Q* factor, finding that it strictly follows $Q\;\propto \;\alpha^{-2}$ (see Figure S2 for other materials), consistent with the typical inverse-square law of symmetry-breaking meta-atoms [43]. This fully confirms that the mode is a typical symmetry-protected BIC.

After monolayer TMDs are integrated onto the metasurface, significant coherent energy exchange occurs between the excitons and the q-BIC cavity mode. For the TMDs, the complex dielectric function can be described by the Lorentz oscillator model [44,45,46,47]:

(2) $$\varepsilon \left(\omega \right)=\varepsilon_{B}+\frac{f_{0}\omega_{0}^{2}}{\omega_{0}^{2}-\omega^{2}-i\omega \gamma_{0}}.$$

Here,${\;\varepsilon }_{B},\;f_{0}$, $\omega_{0}$, and $\gamma_{0}$ represent the background dielectric constant, oscillator strength, Lorentz resonance frequency, and intrinsic damping constant, respectively. Given their atomically thin nature, we approximate the four monolayer TMDs (MoSe_2_, MoS_2_, WS_2_, and WSe_2_) as 1-nm-thick isotropic dielectric films [48]. Despite the inherent optical anisotropy of 2D materials, the isotropic approximation remains valid as the localized q-BIC field predominantly couples to in-plane exciton dipoles, rendering out-of-plane contributions negligible (Figure 1f). Although modeling the sub-nanometer TMD monolayers as 1-nm effective layers ensures numerical stability, this volumetric expansion systematically overestimates the absolute coupling strengths, given that g scales with the square root of the active medium volume ($g\propto \sqrt{V}$). Nevertheless, because this geometric approximation is uniformly applied across all tailored configurations, the relative coupling hierarchy and the comparative trends in polariton dynamics are strictly preserved. The intrinsic parameters of the four monolayer TMD materials used in this study were obtained from references [14,47,49,50,51], and the specific values are detailed in Table S2 of the Supporting Information.

In Figure 2a–d, effective tuning between the cavity mode and exciton energies is achieved by adjusting the asymmetry parameter θ. Clear anticrossing features are observed for MoSe_2_, MoS_2_, WS_2_, and WSe_2_, serving as a prominent spectral signature of strong light–matter coupling. This further demonstrates the broad generalizability of this design strategy across various excitonic systems. Under zero-detuning conditions, we extract the reflection spectra of both the bare cavity and the strongly coupled hybrid systems. These resonance features can be characterized by a Fano-type function $R\left(\omega \right)={\left|r\left(\omega \right)\right|}^{2}$, where the reflection amplitude is expressed as [52,53,54]:

(3) $$r\left(\omega \right)=a_{b}+\sum_{j=1}^{N}\frac{b_{j}\gamma_{j}e^{i\phi_{j}}}{\omega -\omega_{j}+i\gamma_{j}}.$$

Here $a_{b}$, $\phi_{j}$, and $b_{j}$ correspond to the background amplitude, phase, and resonance amplitude parameters of the reflection spectrum, respectively. As shown in Figure 2e–h, the fitting results (dashed lines) are in excellent agreement with the FDTD-simulated spectra (solid lines), indicating that the model accurately describes the spectral response characteristics of the system and provides a reliable basis for the quantitative analysis of exciton–cavity mode coupling behavior. To accurately quantify the interaction strength, the widely used COM is introduced to describe the light–matter interaction process under zero-detuning conditions, thereby yielding the coupling strength and associated dynamic parameters. The second-order COM is given as follows [55]:

(4) $$\hbar \left(\begin{array}{cc} \omega_{Q}-i\gamma_{Q} & g\\ g & \omega_{m}-i\gamma_{m} \end{array}\right)\left(\begin{array}{l} \alpha \\ \beta \end{array}\right)=\hbar \omega_{\pm }\left(\begin{array}{c} \alpha \\ \beta \end{array}\right).$$

Here $\hbar \omega_{Q}$ and $\hbar \omega_{m}$ represent the intrinsic resonance energies of the uncoupled q-BIC mode and the monolayer TMD excitons, respectively. The parameter g characterizes the coupling strength between the two, while α and β are the expansion coefficients of the eigenstates, which satisfy the normalization condition ${\left|\alpha \right|}^{2}+{\left|\beta \right|}^{2}=1$. By diagonalizing Equation (4), the dispersion relation of the hybrid modes can be obtained as follows:

$$E_{\pm }=\hbar \omega_{\pm }=\frac{\hbar }{2}\left[\left(\omega_{Q}+\omega_{m}-i\gamma_{m}-i\gamma_{Q}\right)\right.$$

(5) $$\left.\pm \sqrt{4g^{2}+{\left[\omega_{Q}-\omega_{m}-i\left(\gamma_{m}-\gamma_{Q}\right)\right]}^{2}}\right].$$

Under the zero-detuning condition $\omega_{Q}=\omega_{m}$, the coupling strength ℏg is calculated as:

(6) $$\hbar g=\frac{\hbar }{2}\sqrt{\Omega^{2}+{\left(\gamma_{m}-\gamma_{Q}\right)}^{2}}.$$

Here, Ω denotes the experimentally extracted Rabi splitting frequency. The fitting results in Figure 3 perfectly reproduce the dispersion trajectories extracted from the simulation. To ensure that the hybrid system enters the strong coupling regime, the coupling strength must simultaneously satisfy the following dual criteria:

(7) $$g>\frac{\left|\gamma_{m}-\gamma_{Q}\right|}{2},\;\;g>\sqrt{\frac{\left(\gamma_{Q}^{2}+\gamma_{m}^{2}\right)}{2}}.$$

The former guarantees the existence of Rabi splitting, while the latter ensures that the Rabi splitting can be experimentally verified. The calculated coupling strengths g for the tailored MoSe_2_, WSe_2_, MoS_2_, and WS_2_ systems are 36.8 meV, 23.7 meV, 35.6 meV, and 33.3 meV, respectively. These quantitative results rigorously confirm that all four tailored configurations satisfy the strict threshold requirements for the strong coupling regime. Although minimal asymmetry theoretically yields ultrahigh Q-factors, we deliberately select larger angles to maintain moderate cavity decay rates. This ensures critical linewidth matching with the broad intrinsic linewidths of room-temperature TMD excitons, minimizing the coupling threshold penalty (Equation (7)) and guaranteeing distinct, observable Rabi splitting. While further reducing asymmetry could numerically inflate cooperativity by suppressing cavity loss, the resulting severe linewidth mismatch would collapse the polaritonic branches into unresolvable Fano dips, severely compromising practical far-field modulation performance.

From the COM fitting results in Figure 3, it can be seen that in the four tailored hybrid systems, the extracted data points are in excellent agreement with the theoretical dispersion curves, verifying the accuracy of the COM in describing the dispersion relations of each customized configuration. The uncoupled q-BIC mode ($\omega_{Q}$) and exciton energy ($\omega_{m}$) should have crossed under angular tuning, but the actual upper and lower polariton branches exhibit significant anticrossing behavior and form a clear Rabi splitting ℏΩ at the zero-detuning position (Δ=0). As quantified above, the MoSe_2_ hybrid system exhibits the strongest light–matter coupling, whereas the WSe_2_ configuration shows the weakest. To reveal the physical origin underlying this pronounced contrast among the customized configurations, we comparatively analyze the cavity mode quality factor *Q* and coupling strength g under zero-detuning conditions (Figure 4a). The results show that g does not increase monotonically with *Q*. According to cavity quantum electrodynamics (cQED), the coupling strength g is fundamentally governed by the material’s intrinsic oscillator strength *f* and the localized near-field enhancement |*E*|/|*E*_0_|, which is intrinsically tied to the effective mode volume *V_m_*. As revealed in our near-field analysis (Figure 1f and Figure S1(a-i–c-i)), while the four customized cavities share topologically similar vortex mode profiles, their tailored geometric scaling results in varying peak field enhancements. For instance, the bare cavity tailored for the WS_2_ system is limited by a relatively weaker field enhancement factor (maximum of ~8), whereas the cavity customized for MoSe_2_ benefits from a highly concentrated local field at its resonance. Therefore, the observed variations in coupling strength g among the four systems are the macroscopic manifestation of a synergistic interplay, where the varying near-field confinement capabilities of each tailored q-BIC cavity directly scale the intrinsic exciton transition dipole moments of the specific TMDs [14,49,50,51].

In cQED systems, achieving a large Rabi splitting is only a necessary condition for strong coupling. The practical utility of polaritons crucially depends on their ability to overcome environmental dissipation and maintain the coherent hybrid state. To quantify this capacity, we introduce the cooperativity *C*, which is expressed as [56]:

(8) $$C=\frac{g^{2}}{\gamma_{Q}\gamma_{m}}.$$

Here, g, $\gamma_{Q}$, and $\gamma_{m}$ are the coupling strength, the decay rate of the optical cavity, and the decay rate of the exciton, respectively. This dimensionless parameter essentially quantifies the physical competition between coherent energy exchange and the dual dissipation of light and matter. Combined with the q-BIC linewidths extracted from the Fano fitting in Figure 2e–h, the calculated cooperativity *C* for each system is shown in Figure 4b. A comparison across the four tailored hybrid systems demonstrates that the coherence performance of polaritons does not change monotonically with the coupling strength; instead, it is determined by the competitive mechanism between coupling enhancement and dissipation suppression. Although the tailored WSe_2_ system has the smallest coupling strength g, its significantly reduced photon and exciton dissipation effectively suppresses $\gamma_{Q}{,\gamma }_{m}$, achieving the highest coherence stability (*C* = 3.50). The tailored MoS_2_ system, while maintaining a relatively large g, possesses moderate losses and exhibits sub-optimal cooperativity (*C* = 3.06). In contrast, while the overall dissipation of the WS_2_ hybrid system is not proportionally reduced, its coherent energy exchange rate is severely restricted by the weaker local field enhancement of the customized cavity (Figure S1(c-i)), resulting in a further reduction in cooperativity (*C* = 2.12). Meanwhile, although the MoSe_2_ configuration achieves the maximum coupling strength, the significant intensification of radiative leakage and exciton dissipation substantially increases the dissipation product $\gamma_{Q}\gamma_{m}$, causing its cooperativity to drop to the lowest value (*C* = 2.06) (Table S2). It should be noted that this substantial variation in radiative leakage ($\gamma_{Q}$) across the four systems inherently stems from the distinct geometric scaling required to achieve precise zero-detuning for different exciton transition wavelengths, which inevitably modifies the spatial confinement capability and energy leakage rate of each tailored cavity. Consequently, these findings demonstrate that the specific design of the q-BIC metasurface directly impacts the strong coupling results by simultaneously modulating the near-field confinement and the radiative leakage, which govern g and C, respectively. Thus, achieving robust coherence stability necessitates a synergistic optimization between these design factors, rather than the singular maximization of Rabi splitting.

To reveal the oscillatory coherent energy exchange dynamics between the optical mode and material excitations, we constructed a Heisenberg–Langevin model describing the system dynamics based on open quantum system theory. By solving the corresponding time-domain evolution equations, we obtained the population dynamics of the optical mode and the exciton state, thereby intuitively characterizing the coherent energy exchange mechanism between the two [21,22,23,24,25]. The quantum dynamics of the hybrid system are determined by the total Hamiltonian $\hat{H}$, which can be formally written as:

(9) $$\hat{H}={\hat{H}}_{sys}+{\hat{H}}_{int}+{\hat{H}}_{bath}+{\hat{H}}_{drive}.$$

where the free Hamiltonian of the system and the interaction term are respectively taken as:

$${\hat{H}}_{sys}=\hbar \omega_{Q}{\hat{a}}^{†}\hat{a}+\sum_{i}\hbar \omega_{i}{\hat{\sigma }}_{i}^{+}{\hat{\sigma }}_{i}^{-},$$

(10) $${\hat{H}}_{int}=\sum_{i}\hbar \left(g_{i}{\hat{a}}^{†}{\hat{\sigma }}_{i}^{-}+g_{i}^{*}\hat{a}{\hat{\sigma }}_{i}^{+}\right).$$

The definition of this model is consistent with the treatment of light–matter strong coupling systems in the appendix of [24]. The time evolution of the system operator $\hat{O}\left(t\right)$ is given by the Heisenberg–Langevin equation, which takes the general form:

(11) $$\frac{d}{dt}\hat{O}\left(t\right)=\frac{i}{\hbar }\left[\hat{H},\hat{O}\left(t\right)\right]-\sum_{\mu }\gamma_{\mu }\hat{O}\left(t\right)+{\hat{F}}_{\hat{O}}\left(t\right).$$

Under the rotating wave approximation (RWA) and after eliminating the degrees of freedom of the continuous reservoirs, the effective equations of motion for the optical mode and exciton excitations are obtained:

(12) $$\begin{array}{cc} \dot{\hat{a}} & =-(i\Delta_{Q}+\gamma_{Q})\hat{a}-\sum_{i}(ig_{i}+\gamma_{Qi}){\hat{\sigma }}_{i}^{-}+\sqrt{\Gamma_{Q}}{\hat{A}}_{Q}+{\hat{F}}_{Q}\\ {\dot{\hat{\sigma }}}_{i}^{-} & =-(i\Delta_{i}+\gamma_{i}){\hat{\sigma }}_{i}^{-}-(ig_{i}^{*}+\gamma_{Qi})\hat{a}-{\hat{A}}_{Q}{\hat{\sigma }}_{iz}+{\hat{F}}_{i} \end{array}.$$

In the weak excitation limit, by linearizing the aforementioned operator equations and taking the expectation values, the time-domain population evolution of the system can be numerically solved, which is defined as $n_{Q}(t)=\langle {\hat{a}}^{†}\hat{a}\rangle$ and $n_{i}(t)=\langle {\hat{\sigma }}_{i}^{+}{\hat{\sigma }}_{i}^{-}\rangle$. The obtained results yield the population oscillation frequencies and decay lifetimes, reflecting the polaritonic quantum dynamic behavior jointly determined by coherent energy exchange and overall system dissipation under strong coupling conditions.

The aforementioned physical picture established based on frequency-domain parameters is directly verified in ultrafast time-domain dynamics. Figure 4c–f illustrate the population Rabi oscillations of the four tailored systems under optical excitation, indicating that the energy exchange rate and coherent decay behavior constitute two relatively independent dimensions for describing polariton dynamics. Regarding the oscillation frequency, the population oscillation period increases as the coupling strength g decreases, i.e., T∝1/g. This trend is perfectly consistent with the variation in g in Figure 4a, highlighting the advantage of the MoSe_2_ system in ultrafast energy response. The cooperativity *C* shown in Figure 4b is highly consistent with the decay trends in Figure 4c–f. We found that the tailored MoSe_2_ system exhibits the shortest population oscillation period of 56 fs, while the WSe_2_ configuration is able to maintain its coherent polaritonic exchange for a much longer duration, owing to the maximal suppression of its intrinsic dissipation. These completely self-consistent results in both the frequency and time domains not only intuitively reflect the robust coherence stability of high-cooperativity systems, but also clearly reveal the decoupling between the energy exchange rate and coherence lifetime. This decoupling originates from the geometric zero-detuning engineering required across distinct spectral bands, rather than the intrinsic properties of TMD excitons.

In summary, the light–matter interaction strength and coherence stability do not follow a simple law dominated by a single *Q* factor. Instead, they are governed by a synergistic interplay of multiple factors. Specifically, the intrinsic exciton transition dipole moment and the local field enhancement jointly determine the energy exchange rate, while the overall system dissipation dictates the coherence lifetime. This clear functional decoupling directly maps our customized configurations onto distinct application regimes. On one hand, for tasks prioritizing rapid energy response, such as ultrafast optical switches, the tailored MoSe_2_ configuration stands out by delivering an ultrafast Rabi exchange period of 56 fs. In contrast, applications like polariton condensates and quantum information processing rely heavily on sustained polaritonic coherence. Such systems demand minimal intrinsic dissipation, making the WSe_2_ configuration the optimal choice to maximize cooperativity (*C* = 3.50) and shield polaritonic states against environmental decoherence. Ultimately, this geometry-driven decoupling not only provides clear physical criteria for optimizing 2D material-microcavity systems but also establishes practical design guidelines for tailoring hybrid configurations to diverse polaritonic applications.

## 3. Conclusions

In this work, based on a customized all-dielectric metasurface q-BIC platform, we achieved room-temperature strong coupling with four representative monolayer TMDs (MoSe_2_, MoS_2_, WS_2_, and WSe_2_) in the visible and near-infrared spectral ranges. We systematically evaluated the coupling strength, cooperativity, and time-domain dynamic characteristics of these tailored hybrid systems. Our results demonstrate that precise zero-detuning matching between the cavity mode and distinct exciton transitions can be successfully achieved through the synergistic scaling of structural asymmetry and geometric dimensions. Clear dispersion anticrossing and Rabi splitting were observed across all four customized configurations, confirming the excellent spectral scalability of the q-BIC design framework. Through a comprehensive analysis incorporating the COM and quantum dynamics, we demonstrate that the macroscopic strong coupling behavior is strictly governed by the interplay between the tailored photonic environment (e.g., near-field enhancement and mode volume) and the specific excitonic properties of the TMDs. Specifically, the customized MoSe_2_ hybrid system exhibits an ultrafast Rabi exchange period of 56 fs, demonstrating significant potential for ultrafast active photonic devices, such as all-optical switches. In contrast, the tailored WSe_2_ system effectively mitigates both cavity and exciton decay rates to achieve the highest cooperativity (*C* = 3.50), providing a robust platform for coherence-driven applications including polariton condensation. By elucidating that the decoupling between the energy exchange rate and coherence lifetime is a practical outcome of geometric zero-detuning engineering across diverse spectral regimes, this study establishes a rational design framework for the configuration of room-temperature polaritonic devices, providing valuable guidance for their future implementation in optical processing and quantum information systems.

## Figures and Tables

**Figure 1:**
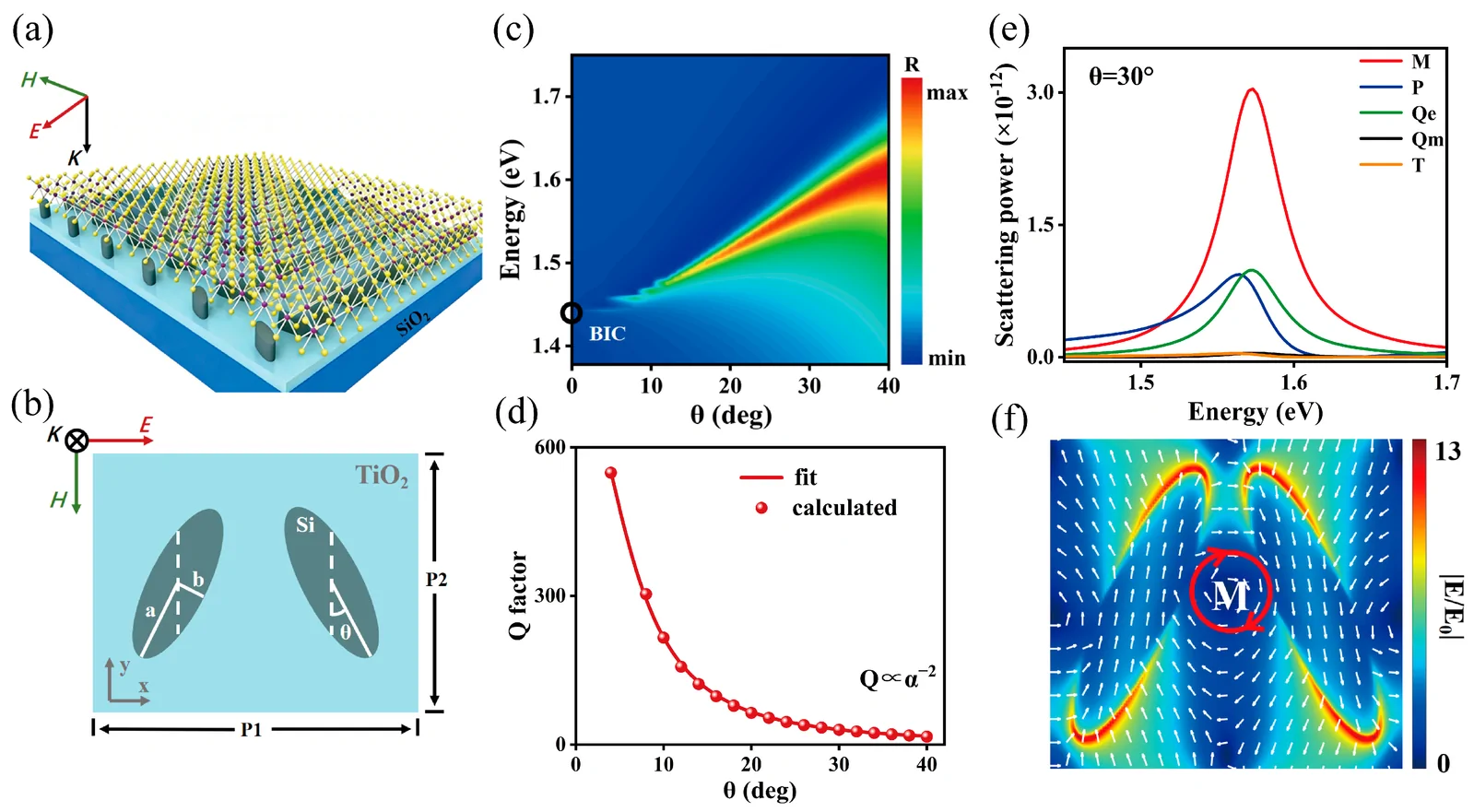
(**a**) Schematic diagram of the strong coupling system between tilted elliptical silicon pillars and monolayer TMDs. (**b**) Cross-sectional view of the unit cell in the *x*–*y* plane. Customized MoSe_2_ resonant cavity (**c**–**f**). (**c**) Reflection color map as a function of the tilt angle θ, with the black circle marking the ideal BIC mode. (**d**) Evolution of the *Q* factor with θ: calculated values (red dots) and fitting curve (red solid line). (**e**) Scattering contributions of different multipole moments in the unit cell for MoSe_2_ at θ = 30°. (**f**) Cross-sectional electric near-field distribution at the resonance peak of the MoSe_2_ cavity.

**Figure 2:**
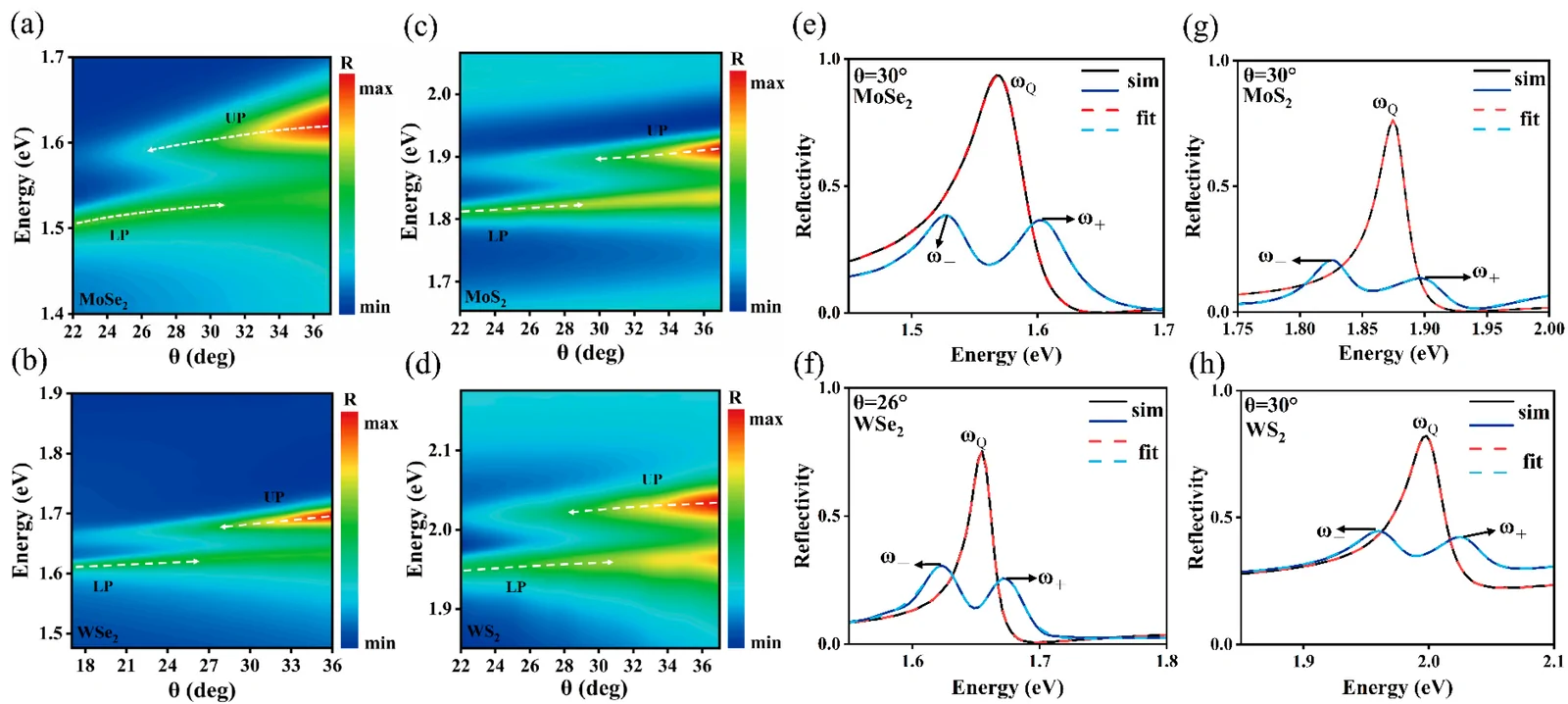
(**a**–**d**) Mapping plots of the reflection spectra as a function of θ for the coupled hybrid systems of MoSe_2_, WSe_2_, MoS_2_, and WS_2_, respectively. (**e**–**h**) q-BIC modes at different peak positions and the fitted (dashed lines) and simulated (solid lines) reflection spectra for strong coupling at zero detuning for MoSe_2_, WSe_2_, MoS_2_, and WS_2_, respectively.

**Figure 3:**
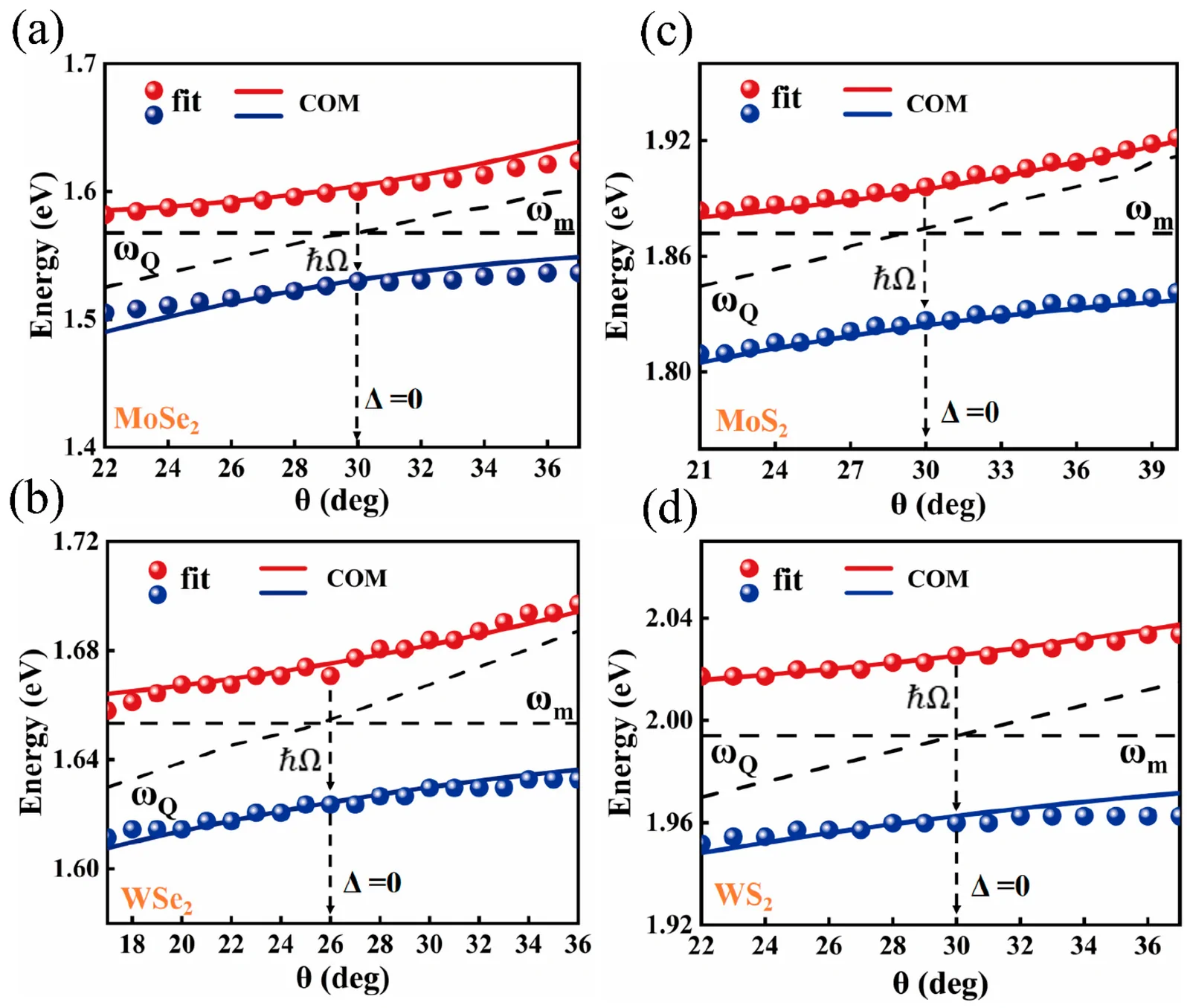
Dispersion relations of the hybrid systems obtained through fitting (blue dots) and the coupled oscillator model (solid lines) after coupling different monolayer TMDs with the resonant cavity: (**a**) MoSe_2_, (**b**) WSe_2_, (**c**) MoS_2_, and (**d**) WS_2_.

**Figure 4:**
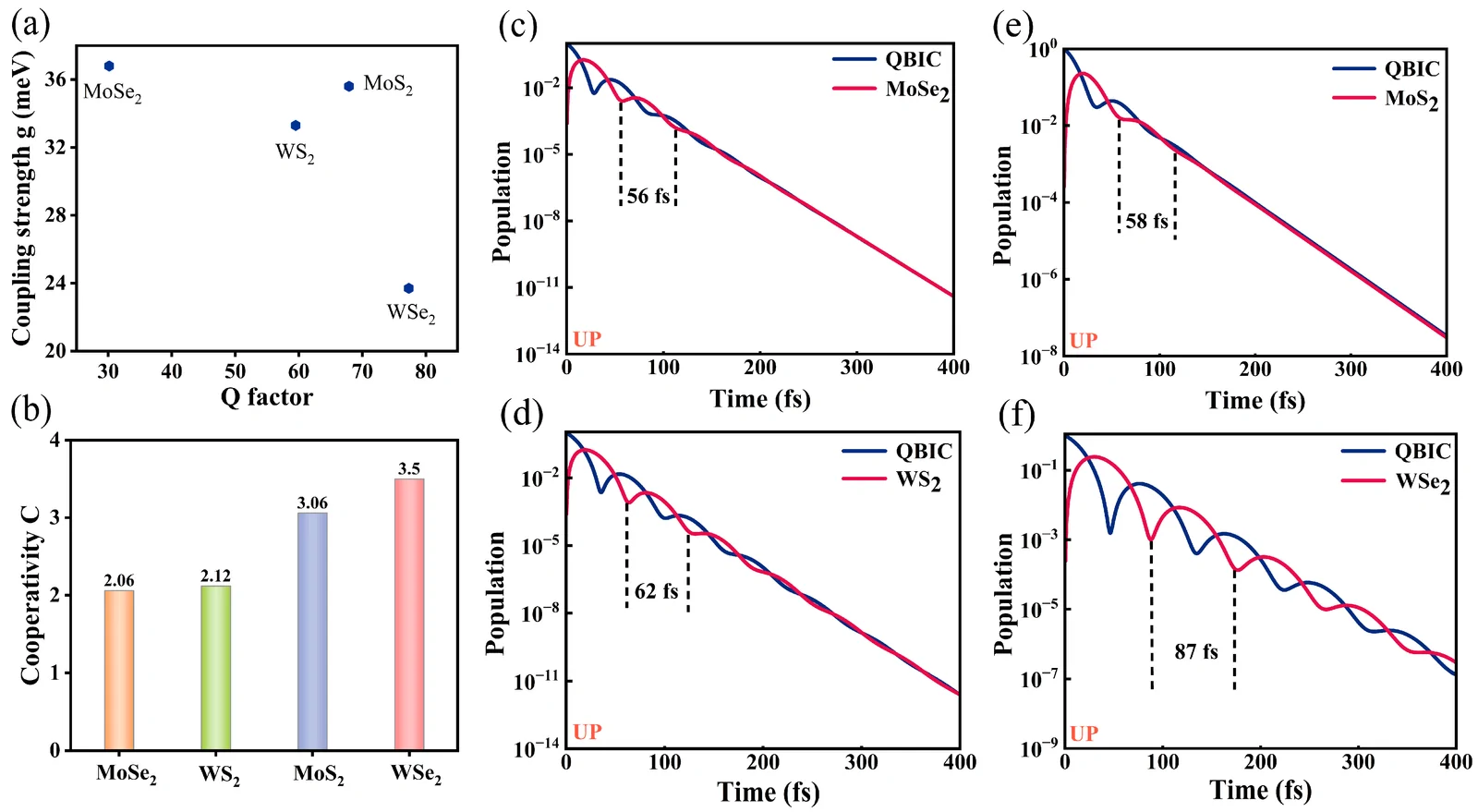
(**a**) Comparison scatter plot of coupling strength g and quality factor *Q*. (**b**) Bar chart of the cooperativity *C* for the four coupled systems. (**c**–**f**) Time-domain evolution of the strongly coupled systems q-BIC mode (blue solid lines) and TMD excitons (pink solid lines): (**c**) MoSe_2_, (**d**) WS_2_, (**e**) MoS_2_, and (**f**) WSe_2_.

## Data Availability

The raw data supporting the conclusions of this article will be made available by the authors on request.

## Supplementary Materials

The following supporting information can be downloaded at: https://www.mdpi.com/article/10.3390/nano16191197/s1, Table S1: Geometric parameters of the silicon elliptical cylinder metasurface; Figure S1: Scattering contributions of different multipoles and spatial distributions of the electric field; Figure S2: Evolution mapping of reflection spectra and variation of the Q factor; Table S2: Exciton parameters and the corresponding bare q-BIC cavity linewidths. References [37,38,57,58,59] are cited in the supplementary materials.
