# Supplementary material for: Energy Exchange and Coherent Behavior of Monolayer TMD Polaritonic Systems in All-Dielectric Metasurfaces Supporting Quasi-Bound States in the Continuum
Source: Nanomaterials (Basel). 2026 Sep 22;16(19):1197. doi: 10.3390/nano16191197 (PMC13666841; doi:10.3390/nano16191197)
Supplement: Supplementary file 1 [file nanomaterials-16-01197-s001.zip › nanomaterials-4551381-supplementary.pdf]

Supporting Information for "Energy Exchange and Coherent Behavior of Monolayer TMD Polaritonic Systems in All-Dielectric Metasurfaces Supporting Quasi-Bound States in the Continuum"

Jiani Li [1,†], Tao Jiang [1,†], Ze Peng [1], Ze Li [1,2] and Meng Wang [1,2,*]

[1] Research Center for Quantum Physics and Technologies, School of Physical Science and Technology, Inner Mongolia University, Hohhot 010021, China; jn-li@mail.imu.edu.cn (J.L.); taojiang@mail.imu.edu.cn (T.J.); p13369362076@outlook.com(Z.P.); li@imu.edu.cn (Z.L.)
[2] Key Laboratory of Semiconductor Photovoltaic Technology and Energy Materials at Universities of Inner Mongolia Autonomous Region, Inner Mongolia University, Hohhot 010021, China
* Correspondence: wangmeng@imu.edu.cn
† These authors contributed equally to this work.

1. Table S1

Table S1 summarizes the geometric parameters of the silicon elliptical cylinder metasurface corresponding to the q-BIC resonant cavity adapted for three monolayer transition metal dichalcogenides (TMDs): WSe_2, WS_2, and MoS_2. These parameters include the tilt angle (θ), lattice periods (P_1 and P_2), semi-major (a) and semi-minor (b) axes of the ellipse, pillar height (h), and the inter-ellipse distance. All structural dimensions have been meticulously optimized to achieve precise spectral matching between the q-BIC cavity mode and the intrinsic exciton resonance energy of each corresponding TMD monolayer.

Target TMDs	Tilt angle θ (deg)	Period P_1 (nm)	Period P_2 (nm)	Semi-major axis a (nm)	Semi-minor axis b (nm)	Height h (nm)	Inter-ellipse distance (nm)
WSe_2	26°	630	520	165	52	60	280
WS_2	30°	420	420	110	45	60	200
MoS_2	30°	570	450	135	42	60	260

2. Multipole Expansion Details

Multipole expansion is widely used in periodic structures to quantitatively analyze the contributions of various electromagnetic multipole components to far-field radiation. By

integrating the induced current density $\boldsymbol{j}$ within the nanostructure, the explicit expressions for each spherical multipole component in the Cartesian coordinate system can be rigorously obtained [37-38]:

$$P_\alpha = \frac{1}{i\omega}\int d^3 r\, j_\alpha, \tag{1}$$

$$M_\alpha = \frac{1}{2c}\int d^3 r\, (\boldsymbol{r}\times\boldsymbol{j})_\alpha, \tag{2}$$

$$T_\alpha = \frac{1}{10c}\int d^3 r\, [(\boldsymbol{r}\cdot\boldsymbol{j})r_\alpha - 2r^2 j_\alpha], \tag{3}$$

$$Q_{\alpha\beta}^{(e)} = \frac{1}{2i\omega}\int d^3 r\, \left[r_\alpha j_\beta + r_\beta j_\alpha - \frac{2}{3}\delta_{\alpha\beta}(\boldsymbol{r}\cdot\boldsymbol{j})\right], \tag{4}$$

$$Q_{\alpha\beta}^{(m)} = \frac{1}{3c}\int d^3 r\, [(\boldsymbol{r}\times\boldsymbol{j})_\alpha r_\beta + (\boldsymbol{r}\times\boldsymbol{j})_\beta r_\alpha]. \tag{5}$$

In the equations, c is the speed of light, ω is the driving frequency, and $\boldsymbol{r}$ is the position vector from the origin to a point in Cartesian coordinates $(\alpha,\beta = x,y,z)$. The terms $P_\alpha, M_\alpha, T_\alpha, Q_{\alpha\beta}^{(e)}$, and $Q_{\alpha\beta}^{(m)}$ correspond to the electric dipole moment, magnetic dipole moment, toroidal dipole moment, electric quadrupole moment, and magnetic quadrupole moment, respectively.

The corresponding far-field radiation intensity for each multipole component is given by [57]:

$$I_P = \frac{2\omega^4}{3c^3}\sum_\alpha |P_\alpha|^2, \tag{6}$$

$$I_M = \frac{2\omega^4}{3c^3}\sum_\alpha |M_\alpha|^2, \tag{7}$$

$$I_T = \frac{2\omega^6}{3c^5}\sum_\alpha |T_\alpha|^2, \tag{8}$$

$$I_{Qe} = \frac{\omega^6}{5c^5}\sum_{\alpha,\beta} |Q_{\alpha,\beta}^{(e)}|^2, \tag{9}$$

$$I_{Qm} = \frac{\omega^6}{40c^5}\sum_{\alpha,\beta} |Q_{\alpha,\beta}^{(m)}|^2. \tag{10}$$

$$I_{total} = I_P + I_M + I_T + I_{Qe} + I_{Qm}. \tag{11}$$

Finally, by normalizing the incident power density, the scattering cross-section contributions of each multipole ($C_{sca}^{P}, C_{sca}^{M}, C_{sca}^{T}, C_{sca}^{Qe}, C_{sca}^{Qm}$) are obtained. Subsequently, the scattering cross-sections of each multipole are summed to obtain the total scattering cross-section of the metasurface system [58-59]. Given that the scattering contribution of the toroidal dipole T is negligibly small in this spectral range, it is omitted in the final summation, yielding:

$$C_{scat}^{total} = C_{sca}^{P} + C_{sca}^{M} + C_{sca}^{Qe} + C_{sca}^{Qm}$$
$$= \frac{4k^4}{3\varepsilon_0 |E_0|^2}\left[\sum_{\alpha}\left(|P_\alpha|^2 + |M_\alpha|^2\right) + \frac{3k^2}{10}\sum_{\alpha,\beta}|Q_{\alpha\beta}^{(e)}|^2 + \frac{3k^2}{80}\sum_{\alpha,\beta}|Q_{\alpha\beta}^{(m)}|^2\right] \tag{12}$$

In the equations, $|E_0|$ is the electric field amplitude of the incident light, $k = \omega/c$ is the wavenumber, and ε_0 is the vacuum permittivity.

3. Figure S1

Figure S1. (a–c) Scattering contributions of different multipoles at the resonance peak positions for the q-BIC resonant cavities corresponding to WSe_2, MoS_2, and WS_2, respectively. (a-i)–(c-i) Spatial distributions of the electric field calculated at the respective resonance wavelengths.

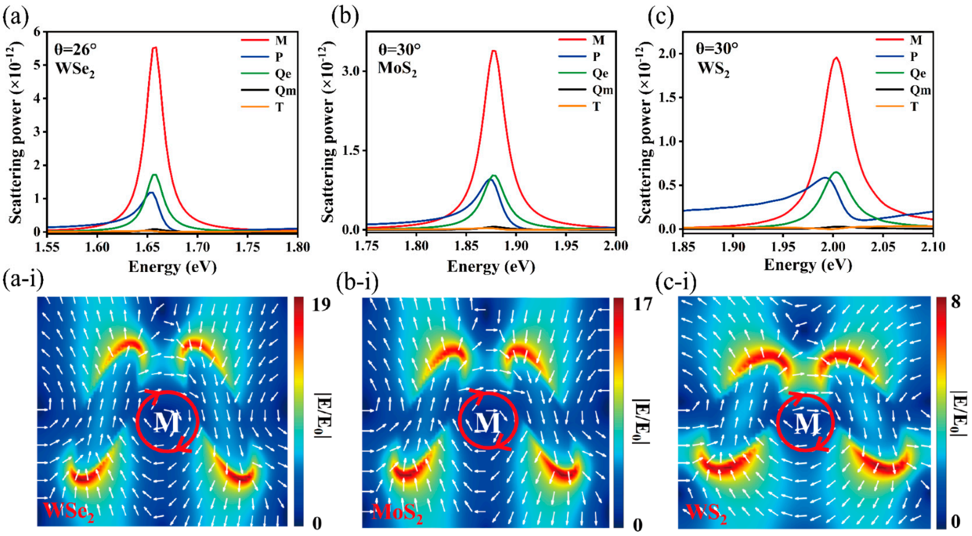

The multipole decomposition results indicate that the resonance response of the metasurface within this spectral range is predominantly governed by the magnetic dipole (*M*). This conclusion is further corroborated by the near-field electric field distribution: the electric field vectors (white arrows) between adjacent elliptical cylinders form a clear, closed circular vortex profile. Such circulating displacement currents are a typical physical hallmark of magnetic dipole resonance in dielectric nanostructures.

4. Figure S2

Figure S2. (a–c) Evolution mapping of reflection spectra as a function of the tilt angle θ for WSe_2, MoS_2, and WS_2, respectively; the black circles mark the ideal BIC modes. (a-i)–(c-i) Variation of the *Q* factor with θ where red dots represent the calculated values and red solid lines represent the fitting curves.

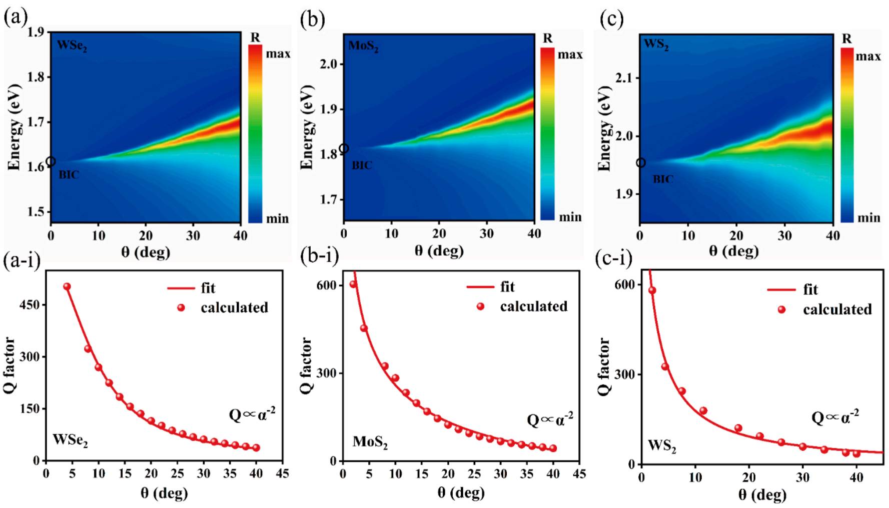

When the tilt angle $\theta = 0°$, the structure maintains high symmetry, and the resonance linewidth completely disappears at the BIC point marked by the black circle. The system is in an ideal bound state in the continuum (BIC) protected by symmetry, preventing radiative coupling with the free-space optical field. As θ increases, the structural symmetry is broken, and the original dark-state BIC mode leaks into a bright-state quasi-bound state in the

continuum (q-BIC), manifested as a gradual broadening of the resonance band in the reflection spectra. Figures S2 (a-i)–(c-i) show the Q factors extracted at different tilt angles. The red solid line fitting results show that the Q factor and the asymmetry parameter α satisfy $Q \propto \alpha^{-2}$, which is the definitive evidence that this system is a typical symmetry-protected BIC.

5. Table S2

Table S2 summarizes the exciton parameters and the corresponding bare q-BIC cavity linewidths for the four transition metal dichalcogenides (TMDs). In our FDTD simulations, each monolayer TMD is modeled as an effective 1-nm-thick dielectric layer. To accurately replicate the predominantly excitonic resonance response within this effective thickness model, the background dielectric constant is uniformly set to $\varepsilon_B = 1.0$ for all materials. The table below lists the remaining specific parameters: oscillator strength f_0, intrinsic exciton transition energy ω_m , exciton loss γ_m , along with the q-BIC resonant cavity loss γ_Q, and light–matter coupling strength g. These parameters were used for the quantitative calculation and analysis of the strong coupling criteria and the polariton Rabi splitting in the main text.

	Oscillator strength f_0	Exciton peak position ω_m (eV)	Exciton γ_m (meV)	BIC resonant cavity γ_Q (meV)	Coupling strength g (meV)
$MoSe_2$	0.6	1.57	26	25.3	36.8
WS_2	0.5	2.0	30	17.4	33.3
MoS_2	0.5	1.87	30	13.8	35.6
WSe_2	0.3	1.65	15	10.7	23.7
